# Critique in context: Pragmatic sociology’s contribution to health research

**DOI:** 10.1177/13634593261474434

**Published:** 2026-08-13

**Authors:** Catarina Delaunay, Luís Gouveia

**Affiliations:** 1 Universidade NOVA de Lisboa, Lisbon, Portugal

**Keywords:** Pragmatic Sociology, Critical Sociology, health and medicine, justification regimes, orders of worth, sociology of critical capacity

## Abstract

Since the 1990s, marked by *Health*’s inaugural issue and the publication of Boltanski and Thévenot’s *De la justification*, the Sociology of Health and Medicine has been shaped by traditions rooted in Critical Sociology, particularly Foucauldian, Marxist, and Bourdieusian paradigms. These frameworks have offered incisive critiques of power, biopolitics, medicalization, and neoliberal reframing of health responsibility. Concurrently, another sociological movement emerged through the development of French Pragmatic Sociology, or the “sociology of critical capacity.” By foregrounding actors’ justifications, plural conceptions of worth, and regimes of engagement, it reorients the analysis from structural domination to situated critique, one that is attuned to the complexity, uncertainty, and ambivalence of health-related action. It enables a relational, context-sensitive approach to the lived experiences of patients and professionals as they engage with institutional arrangements and enact critique within situated action. Drawing on a non-exhaustive, interpretive review of empirical studies engaging with Pragmatic Sociology, particularly in healthcare controversies, clinical decision-making, patient–professional interactions, and health innovation, this article shows how this perspective fosters nuanced understandings of normativity, critique, and coordination in health practices. It traces this theoretical realignment and argues that Pragmatic Sociology offers a compelling, flexible, and still underutilized epistemological and methodological framework for health research, sustaining and extending critique through an empirically grounded analysis.

## Introduction

Over recent decades, rapid transformations in healthcare services and medical practices have generated new objects of inquiry. Health systems are increasingly characterized by distributed forms of expertise, increased technological mediation and plural moral values. Clinical decision-making frequently takes place where evidence is incomplete or contested, as illustrated by precision medicine, assisted reproduction, end-of-life care, rare diseases, antimicrobial stewardship, and the allocation of scarce healthcare resources. Digital health platforms, artificial intelligence, patient-generated data, and online health communities have further multiplied the actors, forms of expertise, and evaluative criteria involved in healthcare. Concurrently, contemporary healthcare places growing emphasis on shared decision-making, patient-centered care, co-production, public and patient involvement, and interdisciplinary collaboration, requiring continuous negotiation between competing conceptions of what constitutes good care, legitimate expertise, fairness, safety, efficiency, autonomy, or quality of life.

These developments raise empirical questions that cannot be fully understood through explanatory frameworks centered primarily on structural domination and reproduction or on governmentality and biopolitics: how actors coordinate action when multiple normative principles are simultaneously legitimate; how professionals and patients justify decisions under uncertainty; how institutional arrangements are continuously negotiated in practice; and how critique itself emerges from everyday situations. It is precisely these questions that have increasingly motivated researchers to engage with Pragmatic Sociology, despite its still limited uptake. By taking actors’ justificatory capacities and conceptions of action seriously, examining how coordination, critique, and commonality are accomplished in concrete situations, and attending to engagements with objects, material environments, and the plurality of human action, with its guarantees and uncertainties, Pragmatic Sociology provides conceptual resources for a richer understanding of contemporary changes in health and medicine. Ultimately, it invites researchers to treat society and the social not as self-evident, but as complex, fluid, and problematic accomplishments.

With this paper, we aim to resituate critique in health through the epistemological and methodological contributions of Pragmatic Sociology, vis-à-vis Foucauldian, Marxist, and Bourdieusian approaches. This comparison intends to engage with some of the most influential critical frameworks, clarifying the distinctive contribution that Pragmatic Sociology can make to the analysis of contemporary health issues. These traditions have been central to the field’s development, though they coexist with other approaches, including those that attend to professional discretion and institutional dynamics (Freidson, 1970), as well as symbolic interactionist approaches emphasizing negotiation and process (Strauss et al., 1985), and phenomenological perspectives on lived experience (Bury, 1982). Thereby, we situate Pragmatic Sociology within a broader continuum of sociological thought spanning structuralist and interactionist perspectives, highlighting both its affinities with and departures from existing approaches.

Scholars working within traditions rooted in Critical Sociology have made major contributions by critically examining issues such as health inequalities, medicalization, biopolitics, and neoliberal responsibility, thereby denaturalizing health and exposing its social construction. In this context, medicalization refers to the process through which non-medical problems become defined and treated as medical issues (Conrad, 2007), while biopolitics captures the governance of populations through techniques oriented to life and health (Foucault, 1978). Similarly, processes of neoliberal responsibilization shift responsibility for health onto individuals through regimes of self-management and risk (Rose, 2007), highlighting how health becomes a site of moral and political regulation.

Yet, although these approaches are heuristically valuable, and some recognize that power operates through active, self-regulating subjects, such that nothing functions without an agentic subject through which to govern (as in Foucault’s conceptualization of governmentality), they often foreground structural dynamics of power and inequality in ways that can underplay the variability of situated action. As a result, these approaches may offer more limited analytical resources for capturing the plural, ambivalent, and negotiated forms through which critique and agency are enacted from within practice.

Concurrently, another sociological movement that emerged and crystallized in the uptake of French Pragmatic Sociology, particularly as developed by Luc Boltanski and Laurent Thévenot, offers an approach to critique grounded in actors’ own justificatory and evaluative capacities. Its key concepts, including orders of worth, regimes of engagement, and reality tests, provide tools for analyzing how plural normative principles are mobilized, contested, and stabilized in practice.

This paper traces the trajectory of this theoretical realignment and conceptual shift from a critical analysis of power over actors to an emphasis on actors’ critical capacities, arguing that Pragmatic Sociology is a productive and analytically distinctive framework that can extend and reorient existing approaches. We begin by briefly mapping the critical legacy in sociological studies of health, acknowledging its strengths while identifying its limitations. We then examine the rise of Pragmatic Sociology in terms of its genealogies and principles, stressing its key differences from critical approaches. Next, we illustrate the empirical productivity of this perspective by reviewing its applications in health research and theorizing its implications for conceptualizing health as plural, critique as situated, and methodology as practice-oriented. Finally, we conclude by showing that Pragmatic Sociology is well suited to addressing complex contemporary health challenges while remaining open to dialogue with other theoretical traditions.

## The critical legacy in the sociology of health and its limits

Critical Sociology has profoundly transformed the sociology of health by demonstrating that health, illness, and healthcare are socially and politically produced rather than natural phenomena. Building on Marxist, Bourdieusian, and Foucauldian traditions, it has exposed the structural determinants of health inequalities, the expansion of medical authority, and the political regulation of bodies and populations. These contributions remain indispensable for understanding power and inequality in health. However, their primary analytical focus on structural domination has often left comparatively less attention to the situated practices through which actors justify, negotiate, contest, and coordinate action in everyday health settings. This section reviews these major contributions while highlighting the dimensions that Pragmatic Sociology seeks to complement.

Building on Marxist and Bourdieusian sociology, scholars demonstrated how class, gender, and race shape illness and access to care, establishing health inequalities as products of social structures rather than biological traits or individual choices (Nettleton, 2020). From a Marxist political economy perspective, particular attention has been devoted to healthcare commodification, neoliberal restructuring of health behaviors, and persistent inequalities, thereby producing a powerful systemic critique of capitalism in health. While some authors emphasized capitalism’s role in producing health inequalities (Navarro, 1976, 2009), others extended Marxist analyses to contemporary healthcare systems and stigma (Scambler, 2012, 2018). Critical Sociology has been especially influential in linking health to large-scale processes of capitalism and neoliberalism. Yet, such approaches privilege meta-narratives of modernity over the plurality of evaluative grammars at work in practice (Wagner, 1999). By emphasizing political-economic explanations and macro-level accounts, they often obscure interactional moments where coordination, justification, and critique unfold. Their focus on singular logics that risk becoming totalizing also leaves comparatively less analytical space for examining how patients, professionals, and organizations negotiate competing normative principles and justify action within concrete situations.

Drawing on Bourdieu’s Field Theory, scholars have shown how health and illness are patterned by habitus, capital, symbolic power, and field dynamics (Collyer et al., 2015; Williams, 1995). Although relational in orientation, this perspective retains a strong structural emphasis, explaining practices primarily through durable dispositions and unequal distributions of capital. The Bourdieusian concept of habitus, though powerful (Williams, 1995), has been faulted, from a pragmatic perspective, for its deterministic character, often portraying practices as overly predetermined at the expense of self-emancipation and creative adaptation (Frère and Jaster, 2024), such as actors’ reflexive and creative capacities to negotiate, criticize, or reconfigure institutional arrangements.

Critical scholarship has likewise demonstrated how everyday experiences and life stages, including childbirth, menopause, hyperactivity, and sexuality, have been redefined as medical problems requiring professional intervention. This work exposed medicine as a social institution of control, revealing the expansion of medical authority into social and moral domains. Conrad’s (2007) account of medicalization consolidated decades of research on how medicine frames deviance and normality, while Clarke et al. (2003) extended this critique through the concept of biomedicalization, emphasizing the technoscientific transformation of bodies, identities, and health practices. Rose (2007) and Rabinow (1999) further analyzed genomics and molecular biopolitics, showing how biological life itself became an object of political intervention. While these analyses convincingly reveal how medical authority expands through broader institutional processes, they devote less attention to how medical categories, technologies, and interventions are interpreted, negotiated, or contested by actors in specific situations.

Foucauldian sociology similarly transformed understandings of health by theorizing biopower, biopolitics, and governmentality, demonstrating how populations are regulated through surveillance, statistics, and norms of bodily conduct. Foucault (2003 [1973], 1978 [1976]) reconceptualized health as a dispositif of normalization while simultaneously emphasizing that governmentality operates through active, self-regulating subjects. Following this, Crawford (1980) identified the moralization of health through healthism, Petersen and Lupton (1996) showed how health promotion constructs “responsible” and “at risk” subjects, and Lupton (1999) analyzed risk as a technology of self-surveillance. While convincingly showing that health institutions exercise disciplinary and normalizing power through psychiatric classifications, public health campaigns, and risk discourses (Hancock, 2018), this persistent focus on domination has been criticized for underestimating resistance, negotiation, and actors’ capacities to justify, question, and reinterpret normative expectations (Boltanski, 2011) in everyday healthcare encounters.

Overall, Critical Sociology has often positioned itself as an “unmasking” science, exposing ideologies that shape actors’ conduct yet remain inaccessible to them, thereby treating their stated reasons for action as “illusions” or “false consciousness” (Celikates, 2006). In health research, disregarding actors’ justificatory capacities is particularly problematic: patients’ accounts of suffering, uncertainty, and moral ambivalence are often treated as epiphenomenal to deeper structures rather than as critical resources in their own right. Pragmatic Sociology addresses these shortcomings, sustaining a commitment to critique, but one that is empirically grounded. It acknowledges actors’ own critical capacities, analyses how legitimacy is accomplished through situated tests and disputes, and examines how coordination emerges across plural evaluative grammars.

## The pragmatic turn

The development of Pragmatic Sociology, particularly through the work of Luc Boltanski and Laurent Thévenot, marks a significant reorientation in sociological approaches to critique, action, and normativity. Rather than grounding critique in external theoretical frameworks, it locates it within social practice by taking seriously actors’ capacities to evaluate, justify, and contest situations. In doing so, it departs from structuralist traditions, especially that of Bourdieu, by moving beyond overdetermined views of social action and the notion of an epistemologically privileged sociologist (Blokker, 2011).

The foundational moment of this approach is Boltanski and Thévenot’s (2006 [1991]) *On Justification*, which introduces the concept of “orders of worth.” Social coordination is understood as relying on plural, historically constituted evaluative frameworks, such as the civic, market, industrial, domestic, and inspired orders, each expressing a distinct conception of the common good. These orders function as publicly available “polities” or moral grammars through which actors classify beings (persons, objects, relations) and justify their actions in situations of dispute. Coordination thus emerges through processes of justification, critique, and compromise, rather than through a single dominant logic. This framework conceptualizes normativity as plural, situational, and testable in practice.

However, the social world cannot be reduced to situations requiring justification, which are situated within a broader context of a public plurality of regimes of action. Boltanski (2012 [1990]) juxtaposes regimes of dispute with regimes of peace, which can be broken down into the regime of justice-justification, the regime of violence, the regime of fairness, and the regime of love or *agape.* Concurrently, Thévenot (1990) broadened the original analytical framework by extending action “under the public,” showing that coordination is not limited to public justification, dispute, or critique, but also involves practical, habitual, and affective relations to the world. In his later synthesis, Thévenot (2006) conceptualizes different “regimes of engagement,” understood as formats for apprehending and engaging with the world, combining cognitive, evaluative, and affective dimensions. They structure how actors categorize relevant elements in a situation and orient action toward specific goods that support coordination. Crucially, these regimes lie along an analytical continuum, ranging from generalized, public forms of coordination to more localized and familiar modes of engagement, including engagement in a plan.

At one pole, the regime of public justification corresponds to situations where actors mobilize orders of worth to justify their actions in reference to shared conceptions of the common good. Here, coordination relies on collective conventions that enable actors to evaluate and rank persons and things according to publicly recognizable criteria. At another level, the regime of engagement in a plan organizes action around predefined objectives, where the environment is functionally configured, and the successful accomplishment of action constitutes the relevant good. Finally, the regime of familiar engagement refers to proximate, embodied relations to the environment, grounded in familiarity, ease, and comfort, where action unfolds without the need for explicit justification.

Across these regimes, action is supported by “investments in forms” (Thévenot, 1984), namely objects, categories, and relational formats that stabilize how situations are apprehended, as well as by corresponding emotional investments, or affective dispositions that render engagement meaningful. The three “grammars of commonality in the plural” further complement this plurality, comprising “plural orders of worth,” “individual choices in a liberal public,” and “personal affinities to multiple common-places,” each grammar organizing distinct ways of communicating and integrating differences (Thévenot, 2014: 18).

In parallel, Boltanski (2011 [2009]) revisits the relationship between critical sociology and the “sociology of critique.” While earlier work emphasized actors’ ordinary critical capacities, this later contribution reintroduces questions of domination, showing how critique can be constrained, displaced, or neutralized by institutional arrangements. Rather than abandoning the pragmatic orientation, Boltanski proposes a double movement: maintaining attention to actors’ capacity for critique while also accounting for asymmetries that shape its conditions of possibility and/or limit its effectiveness. This move addresses a central criticism of early Pragmatic Sociology, namely its alleged neglect of power (Honneth, 2010), by demonstrating how critique operates within, and sometimes against, structured forms of domination.

Together, these developments transform Pragmatic Sociology into a comprehensive framework for analyzing social action. It offers a distinctive conception of critique: not as an external theoretical stance imposed by the sociologist, but as an immanent and situated activity embedded in practice (Barthe et al., 2013). Social life is structured by “tests” (*épreuves*) in which the legitimacy of actions, persons, and arrangements is continuously evaluated, contested, and sometimes stabilized through compromise. Its contribution lies in providing conceptual tools to analyze the plural, situated, and processual character of critique, thereby bridging the gap between structural analysis and interactional dynamics.

## Empirical applications: What Pragmatic Sociology reveals in health

Pragmatic Sociology has increasingly informed research in the sociology of health and medicine by offering a conceptual repertoire capable of capturing how care, expertise, and institutions are coordinated through situated, plural, and morally contested practices. Rather than treating norms as externally imposed or behavior as rule-bound, it foregrounds how actors reflexively compose legitimacy, navigate tensions, and enact critique within concrete situations.

The studies discussed here, identified through a non-systematic but conceptually guided engagement with the literature, span diverse domains, including clinical judgment and decision-making, patient experience, patient–professional interaction or relational care, institutional transformation, and healthcare innovation. We organize them thematically to highlight what they collectively reveal about the moral, epistemic, and organizational dynamics of health.

### Situated judgment under uncertainty and plural evaluative repertoires

A first major contribution of Pragmatic Sociology lies in demonstrating that health-related decisions, whether clinical, organizational, or personal, cannot be reduced to the application of standardized norms or evidence-based rules, but unfold through situated judgments that mobilize plural and often competing evaluative repertoires. This insight is particularly salient in contexts of uncertainty, where actors must navigate incomplete knowledge, conflicting expectations, and institutional constraints.

Moreira’s (2005, 2011) work is central to this perspective. His analysis of clinical guideline development shows that it does not result from a linear translation of scientific evidence into practice but from the coordination of heterogeneous “repertoires of evaluation,” including technical robustness, usability, political acceptability, and methodological adequacy. These repertoires are selectively mobilized and combined to make action possible. In his later work on healthcare rationing, Moreira further demonstrates that allocation decisions are not discrete moments of technocratic calculation but ongoing processes of “pragmatic balancing,” in which clinicians and managers continuously adjust decisions in response to shifting clinical, organizational, and moral demands. Similarly, Delaunay and Gouveia (2024) explore how clinical embryologists navigate a “double pluralism” in decision-making, balancing the “government of life by standards” through “investments in forms” with localized “regimes of engagement” that rely on familiar routines and situational “normative work.” Clinical judgment thus emerges as a situated accomplishment, where actors navigate between standardized evaluative frameworks and locally grounded forms of sense-making. Together, these analyses challenge technocratic models of decision-making, highlighting its temporal, negotiated, and contingent character, and showing how formalized procedures (“investments in forms”) are continually reinterpreted through experiential knowledge and moral evaluation in practice.

This emphasis on ongoing adjustment resonates with Dodier’s earlier notion of “conventional supports of action” (1993), which foregrounds the role of conventions in stabilizing action under uncertainty. He shows that clinical judgment relies on shared evaluative frameworks that provide legitimacy and orientation in ambiguous situations. These supports are not purely cognitive; they are imbued with moral and institutional authority, enabling actors to justify their actions while remaining open to contestation and critique. His earlier work on occupational medicine (Dodier, 1991) illustrates how such conventions are tested when workers’ experiential accounts of illness confront administrative and biomedical classifications. These encounters reveal that legitimacy is not given but negotiated, exposing tensions between lived experience and institutionalized forms of evaluation. Dodier and Camus (1998) extend these insights to the high-pressure context of emergency medicine, showing how professionals oscillate between openness to undifferentiated patient complaints and the need for rapid categorization and intervention. Their analysis of shifting “registers of action” demonstrates that clinical judgment is not merely technical but entails ongoing moral and organizational calibration, emerging as a situated accomplishment shaped by temporal constraints, institutional demands, and ethical considerations.

### Institutional change, justification, and the management of normative pluralism

Extending beyond individual judgment, a second body of work examines how institutions manage normative plurality and competing values, and undergo transformation through processes of justification, compromise, and rearticulation of conventions. Here, Pragmatic Sociology shifts the analytical focus from structures to the ongoing work through which institutional arrangements are stabilized and transformed.

Gagnon and Séguin (2010) show that institutional stability and change are not opposed processes but co-exist and are co-produced through the interplay of multiple orders of worth. Organizations maintain legitimacy by selectively mobilizing, adjusting and recombining justificatory logics, allowing them to adapt without fully abandoning established conventions. Similarly, Dufour et al. (2023) trace how French medical biologists navigate the corporatization of their profession, from entrepreneur to employee, from scientist to manager. This transition involved successive reconfigurations of justificatory logics, from civic to domestic to network, through which actors move as professional roles and identities are redefined, ultimately stabilizing through layered adjustments and compromises rather than through linear change or coherence. This perspective is further extended at a broader scale by Hanrieder (2016), who conceptualizes global health governance as an arena of plural and competing justifications rather than a unified and coherent regime. By mapping how actors mobilize distinct normative orders, such as survival, fairness, production, and spirit, she shows that global health policies are stabilized through fragile and contingent compromises. This analysis challenges monolithic accounts of global health as governed solely by neoliberal or geopolitical logics, instead revealing its inherently plural and contested nature.

Peeters et al. (2023) bring this analysis to the level of organizational design, showing how preventive infrastructures are shaped through multiple forms of justificatory work. Their study of neonatal care units demonstrates how actors translate between different evaluative frameworks, compromise between competing orders of worth, and sometimes attempt to transcend existing valuations. Importantly, these processes are embedded in material arrangements, such as hospital beds, illustrating how negotiated moral and organizational compromises become concretized in devices and infrastructures. These dynamics also extend to professional authority and the negotiation of institutional classifications. Blok (2022) reconceptualizes professional authority as a situational achievement emerging from “jurisdictional engagements,” where actors shift between different modes of coordination, namely public, planned, and familiar, rather than through fixed institutional hierarchies. Nadai and Gonon (2023) similarly demonstrate that work incapacity is not a fixed status or a stable category but rather the outcome of a negotiated classification across competing conventions, stabilized through investments in form, such as legal frameworks, organizational infrastructures, and assessment tools.

### Innovation and the politics of health technologies

A third domain in which Pragmatic Sociology proves particularly fruitful is health innovation, where the moral and political dimensions of valuation, as well as questions of legitimacy and coordination, are central. Here, the framework illuminates how technologies are not simply designed and implemented, but actively constructed through processes of moral and institutional negotiation.

Lehoux et al. (2014) show that health technology development is guided by multiple valuation schemes, including clinical, organizational, economic, and interactional ones, each associated with different regimes of engagement. Developers must align these competing yet interdependent logics to render their innovations legitimate, balancing the promise of improving health with the pursuit of economic value. Innovation thus appears as a process of coordination across heterogeneous evaluative frameworks, revealing that technological development is inherently normative. Kappler (2023) extends this analysis to digital health, demonstrating how e-health infrastructures reconfigure coordination through new forms of measurement, classification, and accountability. While these technologies promise empowerment and personalization, they also introduce opacity and new forms of control by concealing the conventions that underpin them. This ambivalence highlights the dual character of digital health as both enabling and constraining.

Batifoulier (2023) and Da Silva (2023) show how critique itself becomes a resource appropriated within processes of health capitalism and industrialization, where demands for quality, efficiency, or patient-centredness are mobilized to legitimize standardization and marketization, while such reforms may simultaneously reinforce inequalities. Here, critique is not absent but reappropriated, raising questions about its transformative potential. Sharon (2018, 2021) further explores these tensions by examining the “Googlization” of health. She shows how digital platforms mobilize civic ideals of empowerment while embedding market and industrial logics, resulting in a reconfiguration of legitimacy through competing orders of worth. This raises important normative concerns about the subordination of democratic values to commercial imperatives. Similarly, Geampana and Perrotta (2023) show how professionals in fertility care evaluate new technologies through modes of coordination such as efficiency, effectiveness, and patient-centredness. These modes cut across institutional boundaries, revealing shared normative frameworks that structure decision-making.

### Reflexivity, everyday practice, and the situated ethics of care

A further set of studies foregrounds the reflexive and experimental character of everyday health practices, emphasizing how actors actively engage with and transform norms in situ.

Iedema et al. (2020) show how clinicians, through video-assisted reflection, become aware of latent tensions in infection control practices and engage in collective deliberation about appropriate standards. This process reveals that practice is not the application of fixed rules but an ongoing negotiation of what counts as good care. Similarly, Mann (2024) demonstrates that “quality of life” in nephrology is not a stable metric but a plural and evolving value, continuously reinterpreted in practice and enacted differently across clinical situations. Lagerlöf et al. (2024) extend this perspective by showing how primary care managers navigate competing orders of worth when implementing health promotion initiatives. Their analysis highlights how actors must balance immediate care needs, accessibility, and long-term population health goals. Katainen and Gronow (2024) conceptualize habits as plastic, context-sensitive dispositions shaped through experience and interaction, thereby linking individual practices to broader social inequalities and emphasizing the situated and evolving nature of behavior.

### Patient engagement, the fragility of action coordination, and the moral experience of care

A final and particularly rich contribution of Pragmatic Sociology lies in its reconfiguration of critique when examining patient engagement and the moral experience of care, especially in health contexts marked by uncertainty, institutional constraint, and plural normative tensions. Rather than positioning patients as passive recipients or strategic consumers, this body of work foregrounds their capacity to engage in situated forms of evaluation, testing, and contestation. This emphasis on situated critique also intersects with analyses of broader transformations in health systems.

Barbot and Dodier (2002) show how HIV-positive patients navigate multiple forms of expertise by mobilizing critical capacities to test, compare, appropriate, and contest medical claims, demonstrating that critique is enacted within, rather than external to, institutions. Dodier and Barbot (2016) further demonstrate how devices (*dispositifs*) both constrain and enable action (e.g. of patient associations), opening spaces for contestation. In this sense, critique is not external to power but immanent to practical engagement with institutional forms. Romijn’s (2017, 2020) work deepens this perspective by conceptualizing inquietude as a mode of engagement through which patients manage uncertainty or inquietude across consultations, digital searches, and intimate relations in everyday life. He shows how patients strategically modulate their expressions of concern to secure recognition. Consultations thus emerge as sites of justification, where legitimacy is continuously tested and negotiated, and where patients oscillate between vulnerability and agency, while professionals shift between objectification and solicitude.

Portuguese scholarship has significantly extended Pragmatic Sociology into health through a sustained research agenda focused on the delicate, emotionally charged realms of palliative care, assisted reproduction, and psychiatric support. This body of work further enriches this perspective by foregrounding the relational, embodied, and affective dimensions of care. Martins et al. (2022) show that end-of-life conversations are not discrete events but gradual processes of coordination shaped by tacit cues and moral considerations, enabling the alignment of patients’ wishes and professional responsibilities. This perspective is complemented by Martins et al.’s (2018) conceptualization of commonality as a fragile and emergent achievement, continuously negotiated between moments of tension and the stabilizing effects of invested forms. Martins and Delaunay (2016) further highlight the importance of proximity in fostering mutual recognition, showing that professionals’ emotional and moral investments, beyond clinical protocols, are central to legitimizing patients’ voices in end-of-life decisions.

These dynamics are also evident in Delaunay’s (2017, 2018) comparative analysis on palliative care and assisted reproduction, which reveals how communicative breakdowns arise when biomedical language fails to accommodate uncertainty, unmet expectations, and emotional burdens. Such interactions become sites of negotiation and discord, where patients engage in significant moral and affective work, often oscillating between proximity and detachment. Within reproductive medicine, Gouveia and Delaunay (2022) show how patients relate to embryos through shifting dynamics of focusing and unfocusing, attributing multiple and unstable meanings to them, expanding Boltanski’s (2013) grammar of fetal categories. Gouveia et al. (2024) further conceptualize clinical encounters as moral interactions, where patients evaluate professionals through categories such as hospitality and inhospitality, shaping trust and recognition. In parallel, Resende and Carvalho (2024a) examine how moral gestures, understood as acts of recognition and respect, structure everyday sociability in a psychiatric support center, shaping legitimacy, trust, and care within asymmetrical institutional settings. Their complementary study (Resende and Carvalho, 2024b) on passibility explores how relational openness and receptivity, exemplified through painting as gesture, give rise to non-instrumental forms of engagement.

## Extending critique through Pragmatic Sociology

Across diverse settings, ranging from dialysis units and emergency rooms to digital platforms and palliative care, Pragmatic Sociology reveals health as a morally plural and contested field, organized through situated processes of coordination rather than singular logics or hegemonic principles. It offers a compelling framework for analyzing how health is experienced and put to the test in practice, showing how actors navigate uncertainty, mobilize multiple evaluative frameworks, and engage in critique. Health thus emerges as a site of negotiated legitimacy, where meanings are continuously stabilized through the composition of conflicting moral, epistemic, and institutional orders. Nonetheless, these studies highlight tensions, particularly regarding power, inequality, and the limits of compromise, that invite further theoretical development.

The approach has nevertheless attracted some criticism and reassessment, including from Boltanski himself (2011), prompting renewed dialogue with critical theory. One line of critique argues that Pragmatic Sociology merely reproduces actors’ own accounts, thereby losing critical traction (Celikates, 2006), while paying insufficient attention to institutional structures, power dynamics, and historical processes of normative learning that shape social reality (Honneth, 2010). Others contend that its emphasis on local disputes and micro-level justifications leaves macro-structural configurations under-theorized (Wagner, 1999). The framework has also been criticized for appearing “idealistic and overly consensus-oriented,” offering an “overly harmonious picture of the social world” in which social action ultimately rests on justification and compromise (Wagner, 1999).

These criticisms, however, overlook the distinctive conception of critique advanced by Pragmatic Sociology. Rather than applying external normative frameworks, it relocates critique by foregrounding actors’ capacities to interpret and remake social arrangements. From this perspective, critique is not a privileged and detached scientific operation of ideological unmasking, that is, exposing the socio-economic structures, hidden interests, and power relations that shape actors’ thoughts and actions, but an ordinary practice enacted by actors themselves in everyday life (Celikates, 2006). They denounce injustices, mobilize principles of worth, and put institutional arrangements to the test. Central to this perspective are reality tests, in which competing orders of worth confront one another and actors must justify their positions amidst inevitable disagreement. Because disputes constitute a normal and productive feature of social life (Frère and Jaster, 2019), compromises emerge not as stable resolutions but as provisional accommodations open to revision and renewed contestation (Thévenot, 2001). Nor is the framework confined to face-to-face interactions; it extends to economic organizations, political institutions, socio-professional groups, and public issues (Barthe et al., 2013). In this sense, the “sociology of critique” or “sociology of critical capacity” proposes an empirically grounded and plural mode of critique while avoiding the paternalism of top-down ideological unmasking.

Boltanski (2011) further argues that emancipatory critique can emerge from empirical practice without recourse to abstract universalism, while conceptualizing institutions as semantic structures. He likewise distinguishes between “regimes of dispute” and “regimes of peace,” arguing that although the original “orders of worth” described peaceful ways of determining equivalence, other, non-peaceful regimes also exist, including violence (Boltanski, 2012 [1990]). Power, inequality, and domination are not rejected but reframed through attention to how they are enacted, justified, and sometimes contested in concrete situations and practices, as effects of action rather than as its explanatory resources. This situational focus makes visible not only the operations of critique and resistance but also possibilities for negotiation and compromise often obscured in more structuralist paradigms. What results is a mapping of plural evaluative principles that resists reduction to structural determination, capturing both constraint and creativity in organizational life. Applied to health, this reveals patients, caregivers, and professionals actively engaging with power rather than merely being subjected to it.

Later developments have addressed institutional power and capitalist transformation. Boltanski and Chiapello (2005) show how managerial discourse appropriates critique by incorporating new justificatory logics, linking localized justifications to broader power shifts. The framework has also been extended to political ecology (Thévenot et al., 2000), analyzing environmental disputes through plural grammars of the common good. Thévenot (2009) further demonstrates how justificatory grammars circulate and sediment into rules, standards, and policies, indicating that the framework scales from interactional contexts to institutional configurations. Blokker (2011) argues that Pragmatic Sociology bridges agency and structure by recognizing both actors’ moral capacities and the structured nature of knowledge (e.g. plurality of “orders of worth”). Hanrieder (2016) provides a powerful example of this scalar extension, demonstrating how justification practices in global health governance both reflect and reshape institutional orders.

Health, then, becomes a rich terrain for observing immanent critique and the political traction of pragmatic inquiry. Far from being normatively neutral, Pragmatic Sociology exposes the moral and political force of patient-led critique, grounded in situated practices of justification. Inequality and domination are not overlooked but analyzed as outcomes of situated tests and contingent compromises. In their study of HIV clinical trials, Barbot and Dodier (2002) highlight enduring asymmetries of expertise between patients and professionals, yet they also show how such hierarchies are tested and sometimes renegotiated in practice.

This sensitivity to plural and intersecting disputes is particularly valuable in health, where controversies over end-of-life care, public health interventions, or clinical trials are rarely reducible to a single axis of conflict. Health controversies often stem from breakdowns in coordination that generate new forms of critique and reordering. Empirical studies corroborate this. Dodier (1993) shows how AIDS controversies resisted stable compromise, requiring the elaboration of new justificatory principles. Likewise, Moreira (2011) conceptualizes health care rationing not as rule-based allocation but as a situated, context-sensitive practice. Decisions are continuously negotiated amid competing demands and shifting circumstances. Pragmatic Sociology thus does not deny conflict but offers a vocabulary for tracing its situated dynamics and normative stakes.

That said, another line of critique argues that Pragmatic Sociology risks implying an overly egalitarian view of social relations by focusing on actors’ claims to equality; however, this stems from a misunderstanding, since “polities” are not empirical descriptions but ideal constructions (based on the principles of “common humanity” and “common dignity”) that actors mobilize to criticize inequality, whose analytical value lies precisely in contexts marked by asymmetry and domination (Barthe et al., 2013). The principle of symmetry requires recognizing that ordinary actors possess the same fundamental critical and judgmental capacities as social scientists, rejecting any inherent epistemic hierarchy between them (Celikates, 2006).

A third critique may concern the empirical operationalization of its concepts, namely the lack of exhaustiveness and French exceptionalism (Wagner, 1999). The application of “orders of worth” is often dismissed as overly typological and typical of French society, with little relevance to socio-political life in other cultures. However, Boltanski and Thévenot (2006 [1991]) emphasize that the repertoire of orders is open-ended and empirically extensible. New grammars of worth, such as the environmental order (Thévenot et al., 2000) or the network order (Boltanski and Chiapello, 2005), can emerge, as can new regimes, for example “explorative engagement” (Auray, 2011). Additionally, comparative research showed that similar “orders of worth” are mobilized in other contexts, such as American political and social life, albeit with different cultural preferences (Thévenot et al., 2000). Far from confirming its critics, Pragmatic Sociology turns out to be a dynamic and evolving framework (Barthe et al., 2013) that sustains critique while remaining attentive to the plural, ambivalent, and contested nature of health worlds.

Health research illustrates this adaptability, extending the model by identifying new evaluative grammars. For instance, Lehoux et al. (2014) introduce a tension between “health” and “wealth” in technology development, while Sharon (2018) reveals how digital health is legitimated through a mix of civic, industrial, and market-based justifications, suggesting the emergence of a “healthist” or “vitalist” order of worth. Other studies have explored categories like *hospitality* (Gouveia et al., 2024) or *inquietude* (Romijn, 2020), enriching the framework with new empirical concepts. Moreover, authors have productively combined Pragmatic Sociology with other theoretical traditions, for instance, integrating Honneth’s theory of recognition (Martins and Delaunay, 2016), Walzer’s theory of justice (Sharon, 2021), Weber’s ideal types of social action and rationality (Delaunay and Gouveia, 2024), or Law’s concept of ordering (Lagerlöf et al., 2024), thereby enhancing its critical and normative range.

Furthermore, some scholars have explored the intersections and tensions between critical and pragmatic sociologies. Susen (2015) argues that, despite key differences, Bourdieu’s critical sociology and Boltanski’s contribution to pragmatic sociology share important common ground and can be productively integrated to develop a more comprehensive and normatively engaged social theory. Frère and Jaster (2024) advocate a constructive critical sociology that, through the concept of the *social-mien*, empowers actors as co-creators of emancipatory practices, shifting critique from denunciation toward collaborative, processual experimentation.

Viewed epistemologically, Pragmatic Sociology conceptualizes health as a plural and entangled domain in which values coexist, frequently conflict, and remain open to contestation and coordination. Its methodological implications are equally significant. By privileging ethnography and interactional analysis, it directs attention to tests, disputes, and justificatory repertoires as tools for examining normative life in practice (Carlsen et al., 2025). The result is a richer account of ambivalence and compromise than approaches organized primarily around the domination-resistance binary.

Critique, ultimately, is redefined as a situated and socially embedded activity rather than denunciation from above, one that connects the micro-dynamics of justification with macro-level transformations (Barthe et al., 2013) and keeps sociology critical while remaining attuned to actors’ perspectives. It thereby illuminates not only how critique is enacted in specific situations but also how it may be co-opted, constrained, or rendered invisible within macrosocial configurations. Above all, what emerges is the irreducible plurality and normative texture of health: a domain where care is always moral and justification always a collective achievement.

## Conclusion

Pragmatic Sociology offers a heuristically useful framework for researching health, breaking with structural determinism while maintaining a commitment to critique that bridges situational dynamics and structural realities. It is especially well suited to addressing complex contemporary health challenges, while remaining open to dialogue with structural, feminist, and phenomenological approaches.

In fact, contemporary medicine, in fields such as assisted reproduction and precision medicine, increasingly relies on decisions that cannot be reduced to the application of evidence or institutional rules alone. The governance of emerging health technologies requires professionals, patients, relatives, managers, regulators, and technologies to coordinate action across competing evaluative principles. Pragmatic Sociology offers conceptual tools, including orders of worth, regimes of engagement, investments in forms, tests, compromises, and critical capacities, for analyzing how these actors justify decisions, negotiate disagreements, stabilize temporary settlements, and contest existing arrangements without assuming that a single explanatory logic necessarily predominates.

Future research might also employ a Pragmatic Sociology lens to examine contemporary transformations in digital health, where clinicians are increasingly required to integrate new sociotechnical artifacts, such as AI-powered diagnostics, into their work, reconfiguring professional–patient relationships, and renegotiating the boundaries of expertise as algorithms generate knowledge that reinforces, complements, or challenges clinical judgment. Pragmatic Sociology enables researchers to investigate how competing normative grammars are mobilized in practice, how different actors evaluate technological decisions, and how new conventions become institutionalized or challenged through everyday interactions.

Likewise, the growing emphasis on shared decision-making and patient involvement invites investigation into how different forms of expertise become mutually recognizable, how legitimacy is negotiated between experiential and biomedical knowledge, and how disagreements over autonomy, responsibility, and vulnerability are coordinated in concrete situations.

Pragmatic Sociology shows that it is far from normatively weak, blind to power, consensus-biased, methodologically rigid, or politically narrow. On the contrary, it grounds critique empirically in the ordinary capacities of patients, professionals, and activists; illuminates how inequalities are enacted, resisted, and reconfigured in practice; attends to conflict and the fragility of compromises in health controversies; encourages the discovery of new evaluative grammars in response to empirical novelty; and extends from micro-level clinical encounters to global health institutions and capitalist transformations. Rather than advocating a paradigmatic replacement, we present Pragmatic Sociology as part of a broader movement toward a plural and “post-paradigmatic” sociology (Olson et al., 2020; Pernecky, 2016). The question, therefore, is not which paradigm should prevail, but which conceptual tools are most appropriate to the research problem at hand.
